# Optimized Combination of Strength and Electrical Conductivity of Al-Mg-Si Alloy Processed by ECAP with Two-Step Temperature

**DOI:** 10.3390/ma13071511

**Published:** 2020-03-26

**Authors:** Nannan Zhao, Chunyan Ban, Hongfei Wang, Jianzhong Cui

**Affiliations:** Key Laboratory of Electromagnetic Processing of Materials, Ministry of Education, Northeastern University, Shenyang 110819, China; nf1234567@sina.com (N.Z.); wanghf_neu@163.com (H.W.); jzcui@epm.neu.edu.cn (J.C.)

**Keywords:** aluminum alloy, severe plastic deformation, precipitation, strength, electrical conductivity

## Abstract

The mechanical properties and electrical conductivity of 6063 aluminum alloy subjected to equal-channel angular press (ECAP) at room temperature (RT), 200 °C, and two-step temperature schedule (TST) have been investigated in this study. The TST refers to one pass at 200 °C followed by further successive pressing at RT. It is shown that this method is effective in obtaining the combination of high strength and electrical conductivity. After two passes, the higher strength can be achieved in TST condition (328 MPa yield strength and 331 MPa ultimate tensile strength), where the changing parameter is processing temperature from the first pass at 200 °C to the second pass at RT, as compared to two passes in RT condition (241 MPa yield strength and 250 MPa ultimate tensile strength) and two passes in 200 °C condition (239 MPa yield strength and 258 MPa ultimate tensile strength). This performance could be associated with grain refinement and nanosized precipitates in TST condition. Moreover, in contrast to RT condition, a higher electrical conductivity was observed in TST condition. It reveals that high strength and electrical conductivity of 6063 aluminum alloy can be obtained simultaneously by ECAP processing in TST condition because of ultrafine-grained microstructure and nanosized precipitates.

## 1. Introduction

Severe plastic deformation (SPD) techniques, such as equal-channel angular pressing (ECAP) and high-pressure torsion (HPT), are effective in refining grain size down to submicron-meter or nanometer range through inducing intense shear strain, and hence lead to high strength [1]. A reduction of grain size results in a significant increase in strength according to the Hall–Petch relationship at low temperatures [2,3,4]. Pure metals and non-heat-treatable alloys in ultrafine-grained (UFG) structure through SPD processing can obtain high strength, which could be associated with grain refinement and high dislocation density [4,5]. For heat-treatable alloys, precipitation hardening plays a key role in a further enhancement in strength. The combination effect of grain refinement, dislocation entanglements, and precipitations on the strength has attracted considerable attention. In addition, the amount of strain and temperature are important SPD parameters to determine grain refinement, solute distribution, deformation-induced precipitation and mechanical properties [6]. It has been observed that increasing processing temperature leads to an increase in grain size [7,8]. However, dynamic precipitation assisted by high density of dislocations and vacancies during SPD processing at elevated temperature can impede coarsening of grains [9,10]. Most previously reported studies on SPD of heat-treatable alloys usually employ an invariable temperature during consecutive SPD processing, and it is a lack of research on the effect of two-step temperature during processing on the microstructure and strength.

Al-Mg-Si alloys are widely used in electrical engineering because of their high strength-to-density ratio combined with high corrosion resistance, relatively high intrinsic conductivity and low cost compared to copper [7]. It is desired to obtain high strength and excellent electricity conductivity for demanding applications on conductors for overhead power line and conductor rail [11]. However, strength and electrical conductivity are usually mutually exclusive in metallic conductive materials. To improve balance of the strength and electrical conductivity of Al-Mg-Si alloys, some attempts have been made on the base of conventional cold-deformation and artificial aging [12,13,14]. For example, Lin et al. [13] investigated the effect of pre-aging and re-aging on the strength and conductivity of an Al-Mg-Si alloy, and observed that electrical conductivity improved to 58.9% IACS (International Annealed Copper Standard) while the tensile strength was 301 MPa. It should be noted that the strain introduced by these methods is not as high as that introduced by SPD technique. Grain refinement by adopting SPD technique is an effective method of improving strength. At the same time, grain boundaries could be considered to be the least efficient lattice defects scattering electrons in metals and hence provide the smallest negative effect on electrical property compared to solute atoms and second-phase precipitates [15,16]. A further increase of strength of Al-Mg-Si alloys while keeping acceptable electricity property have been investigated by thermo-mechanical processing comprising SPD methods [17]. The strength of Al-Mg-Si alloys can be improved by ECAP processing and subsequent aging without degradation of electrical conductivity. However, controlling strength through this method is very challenging. It is due to a concomitant recovery of UFG microstructure and grain growth during static aging treatment, which can lead to a decrease in strength. Nano-scale precipitates play a key role in improvement of the strength and electrical conductivity simultaneously [7,13,18]. Another way was attempted by introducing dynamic aging through SPD processing at temperatures typically about 100–200 °C, where a classical precipitation treatment is carried out [19,20]. However, the method may be further modified due to low strength when SPD were carried out at constant elevated temperatures.

In the present study, two-step temperature schedule (TST) during consecutive ECAP processing was carried out to develop this approach. The aim of this study is to make low alloying conductive Al alloys show excellent mechanical properties and enhanced electrical conductivity, and to explore its widespread application in wire and cable industry. Thus, a commercial Al6063 alloy is selected as the researched alloy. In addition, the microstructures, mechanical and electrical properties of the alloy processed in RT, 200 °C and TST conditions were investigated in detail.

## 2. Materials and Methods

**Figure 1:**
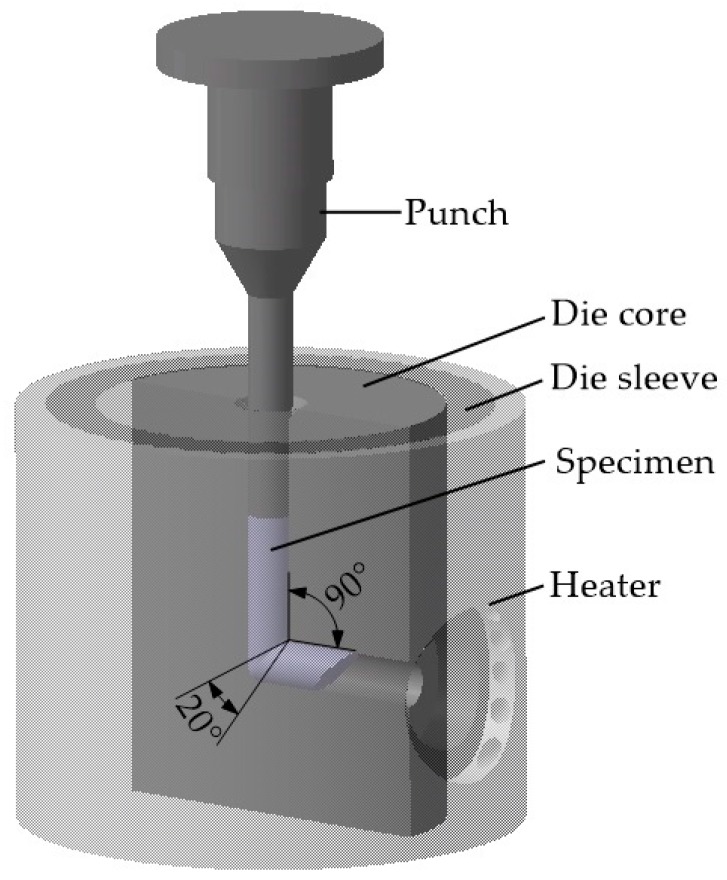
Schematic representation of press die assembly.

The composition in wt.% of the 6063 alloy is Al-0.52Mg-0.49Si-0.09Fe-0.01Ti. Cylindrical billets were cut with 70 mm long and the diameter of 15 mm and were annealed at 560 °C for 1 h followed by immediately quenching in ice water. This is designated the as-annealed condition. These billets were subjected to SPD processing by ECAP using a die with the specification of inner corner angle of 20° and outer corner angle of 90° (as shown in Figure 1), which lead to an imposed strain of ~1.05 per pass. The deformation was carried out with route Bc by a rate of about 1.2 mm/s. The samples were processed up to eight passes at three temperature regimes: room temperature (RT), 200 °C and TST schedule. For RT and 200 °C conditions, the processing temperature is invariable during successive pressing. For the TST condition, billets were processed through only one pass at 200 °C followed by pressing up to seven passes at RT. For elevated temperature processing, the die was heated to required temperature by heating jacket of resistance wire and then billets were held in entrance channel of the die without deformation during ~6 min to stabilize their temperatures. After each pass of ECAP processing, the billets were removed from die and quenched into water immediately for further processing.

Mechanical properties were evaluated by hardness measurements and tensile tests. The Vickers hardness was measured with a FM-700 device (FUTURE-TECH, Kawasaki, Japan) with a load of 100 g and a dwell time of 10 s. The hardness value on each sample was given by the average of at least 8 points. The tensile tests were performed on the universal tensile testing machine AG-Xplus 100 kN (SHMADZU, Kyoto, Japan) at RT, with the strain rate of 0.93 × 10^−3^ s^−1^. The plate tensile samples were machined according to ASTM E8. The cross-section and gauge length of tensile samples were 3 mm × 2 mm and 12.5 mm, respectively. Tensile direction was parallel to extrusion direction (ED), which is the longitudinal direction (LD). Before tensile testing, the species first were cut from billets by using wire-cut electric discharge machine, and then ground by SiC sand paper to remove the oxidation layer. The yield strength was calculated using the 0.2% offset method from the engineering stress-strain curve. Electrical conductivity was measured at RT using the SIGMASCOPE SMP10 (Helmut Fischer, Sindelfingen, Germany) at 60 KHz. The apparatus calibration was conducted at RT before measuring. The electrical conductivity reported in the study was an average of at least ten measurements. The probed zone was about 12 mm in the diameter. The unit of electrical conductivity is %IACS (International Annealed Copper Standard).

The microstructures of as-annealed and processed billets were characterized through optical microscopy and transmission electron microscopy (TEM). Samples were extracted from center region of processed billets and parallel to ED, and then ground and polished following the standard produce. The samples were etched by a solution of 5 g HFB acid and 200 mL H_2_O. For TEM observation, discs of 1 mm thick were cut along LD, and ground to a thickness of 100 µm. Then these discs were thinned by a twin-jet electropolishing unit using a solution of 30% nitric acid and 70% methanol at −30 °C and 25 V. TEM inspections were carried out on a TECNAI G^2^ 20 microscope (FEI, Hillsboro, OR, USA) operating at 200 kV. Representative structure and relative selected area electron diffraction (SAED) pattern were obtained in each condition. Furthermore, the average (sub)grain structure size was quantitatively measured by at least 300 values. Differential scanning calorimetry (DSC) tests were performed on the as-annealed and processed samples using a TA Q100 (TA Instruments, New Castle, DE, USA) under an argon atmosphere with a flow rate of 50 mL/min. In DSC tests, each sample with 40 mg was heated continuously from 100 °C to 500 °C at heating rate of 10 °C/min.

## 3. Results

### 3.1. Microstructural Evolotion during ECAP Processing

**Figure 2:**
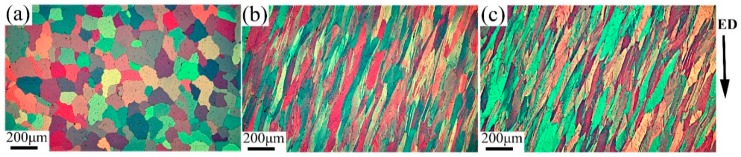
Light-optical micrographs of (**a**) as-annealed samples before ECAP; (**b**) sample processed after one pass at RT and (**c**) at 200 °C.

Figure 2 shows the characterization microstructures of as-annealed and processed 6063 aluminum alloy through one pass at different temperatures. The microstructure of as-annealed sample (Figure 2a) consists of almost equiaxed grains with an average grain size of ~177 µm. The imaging of processed samples shows that the original equiaxed grains are elongated roughly along shear direction due to severe shear deformation (Figure 2b,c). Average width of these elongated grains in samples processed by one pass at RT and 200 °C are ~56 µm and ~61 µm, respectively. Some substructures occur in the deformed samples, such as slip bands and deformation bands derived from crystallographic slip, and shear bands derived from locational deformation. It is noted that the shear bands have an angle of about 45° with the ED, also being parallel to shear direction.

**Figure 3:**
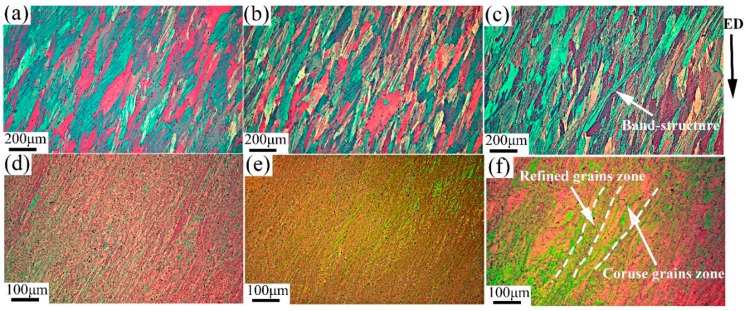
Light-optical micrographs of samples processed (**a**) after two passes on TST condition, (**b**) on RT condition, and (**c**) on 200 °C condition; and (**d**) after eight passes on TST condition, (**e**) on RT condition, and (**f**) on 200 °C condition.

Figure 3 depicts the microstructures of 6063 alloy processed by two passes and eight passes under different conditions. Further successive processing leads to the similar microstructure refinement for samples processed on both TST and RT conditions: (1) the initial coarse grains were gradually fragmented as increasing of pass numbers; (2) the orientation contrast of shear bands and extent of distortion at crossed boundary are improved, confirming that the intensity of shear bands increase with increasing pass number. However, by careful observation of microstructures of samples processed after two passes on RT and TST conditions (Figure 3a,b), it is interesting noting that shear bands are more prevalent in the case of TST condition, and coarse-grained regions still present. These coarse grains are mainly related to their unfavorable grain orientation for dislocations slip, and ECAP processing may lead to existence of higher density of shear bands as a form of the plastic instability. Compared with microstructures on RT and TST conditions, some unique band-like refined zones exist for the sample processed by two passes at 200 °C (as marked in Figure 3c). These band-like structures are consisted of elongated grains. After eight passes (Figure 3d–f) the initial grain boundaries have been hardly recognized, and intersection action between high density of shear bands and deformation bands occur in processed samples. It could be responsible for the grain subdivision and formation of submicron grains with new high angle boundaries [21,22]. In contrast, microstructure with alternate bands between refined grain zone and coarse grains zone are introduced in sample processed by eight passes at 200 °C. Processing at 200 °C leads to more inhomogeneous microstructures than those on RT and TST conditions.

**Figure 4:**
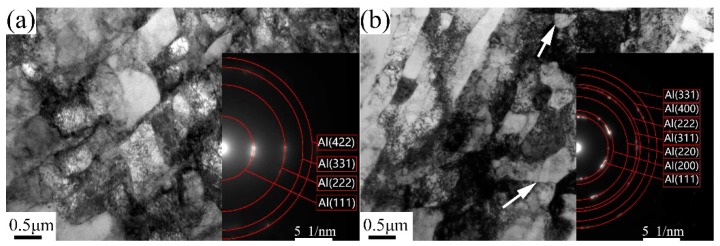
TEM micrographs and corresponding SAED pattern of samples processed after one pass (**a**) at RT and (**b**) 200 °C.

**Figure 5:**
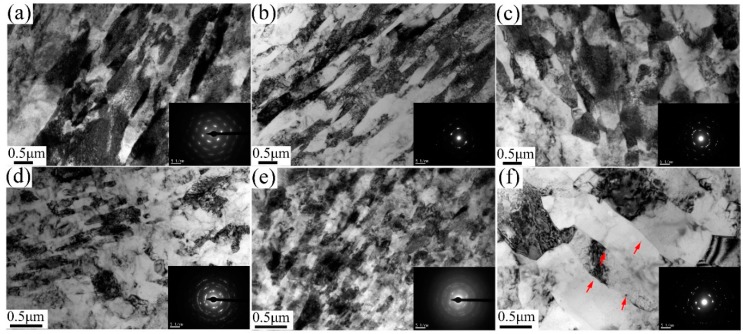
TEM micrographs and corresponding SAED pattern of samples processed (**a**) after two passes on TST condition, (**b**) on RT condition, and (**c**) on 200 °C condition; and (**d**) after eight passes on TST condition, (**e**) on RT condition, and (**f**) on 200 °C condition.

Figure 4 exhibits TEM micrographs of 6063 alloy processed by one pass at RT and 200 °C, and corresponding SAED patterns are inserted in each condition. After one pass at RT, dominant microstructure consists of parallel lamellar boundaries with elongated subgrains of 0.68 ± 0.2 µm in width. These lamellar boundaries are similar to dense dislocation walls (DDW) and enclose dislocation cells. However, after one pass at 200 °C, some equiaxed subgrains develop with average width of ~0.42 µm. It is apparent that the boundaries between elongated subgrains of sample processed by one pass at 200 °C are quite sharper than those in RT condition due to the fact that high processing temperature promotes the dynamic recovery effect. Analysis of SAED patterns (inserted in Figure 4) reveals the SAED pattern consists of some discrete spots showing that subgrain boundaries mostly exhibit low misorientation. Figure 5 presents the TEM micrographs and corresponding SAED patterns of 6063 alloy processed through two and eight passes under different conditions. After two passes, the elongated (sub)grains with defined boundaries develop in samples processed in three conditions. The averaged width of (sub)grains for the samples processed by two passes on RT, TST and 200 °C conditions are 0.23 ± 0.05 µm, 0.39 ± 0.08 µm and 0.51 ± 0.08 µm, respectively. After eight passes, processing at RT and TST conditions produce a mixture of elongated and essentially equiaxed (sub)grains with moderate or high angle boundary, as inferred from rings of diffraction spots in SAED pattern. The averaged width of (sub)grains for sample processed by eight passes at RT and TST conditions is 0.08 ± 0.01 µm. By contrast, processing at 200 °C produces elongated (sub)grains with average width of 0.6 ± 0.1 µm and with low misorientation angle boundaries, as inferred from slightly diffused SAED pattern. A few small equiaxed subgrains with ~0.16 µm are also observed at triple points of three elongated subgrains. Sample processed by eight passes at 200 °C condition has coarser (sub)grains than those at the other two conditions.

### 3.2. Precipitation during ECAP Processing

**Figure 6:**
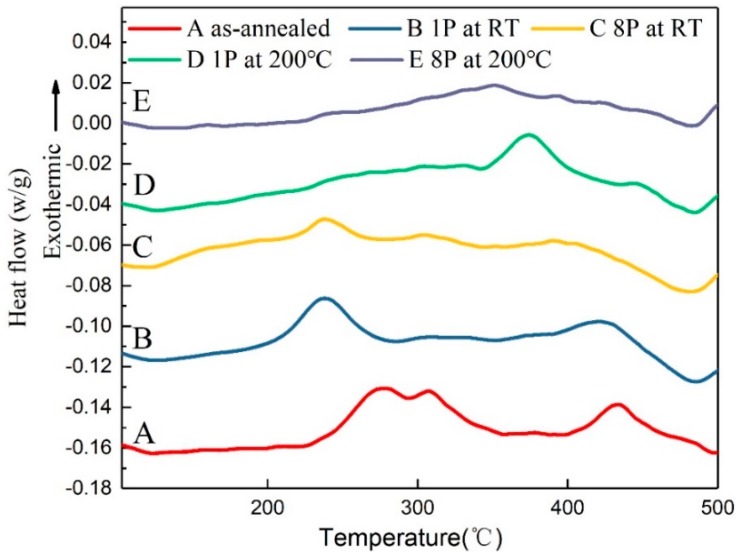
DSC curves of as-annealed and processed samples at RT and 200 °C conditions.

DSC plots are shown in Figure 6 for as-annealed and processed 6063 alloy after one pass and eight passes at RT and 200 °C. The as-annealed alloy exhibits three exothermic peaks in the examined temperature range. The first two exothermic peaks overlap into a broad peak ranged from 232 °C to 348 °C, which represents the successive formation of unstable precipitates β’’and β’. The third exothermic peak ranged of 400 °C to 500 °C represents formation of stable phase β. The reaction peaks appeared in the present study are similar to those reported in similar alloys [23,24]. In the case of RT condition, three exothermic peaks for precipitates formation are also observed. However, it is noted that these peaks shift towards lower temperatures with increasing pass numbers at RT condition, especially for the peak represented of β phase. It confirms that SPD accelerates formation activation of precipitate during subsequent annealing by reducing its activation barrier [23]. This fast aging behavior is probably favored by the high density of defects introduced by severe deformation, which can act as high diffusion path for solute atoms [23] and provide the large number of nucleation sites [7]. For the sample processed by one pass at 200 °C, the intensity of peak ranged from 220 °C to 320 °C decrease in comparison with that in relation to formation of β’’and β’ at RT condition (curve D vs. curve B). It is expected that the observed peak is due to formation of β’’and β’. This is consistent with the fact that pronounced fine β’’and β’ have already formed during one-pass processing at 200 °C, as indicated in following TEM micrographs, which leads to a decrease in these precipitations during subsequent DSC study [25,26]. In the case of sample processed through eight passes at 200 °C, the absence of prominent peaks for formation of β’’ and β’are observed, but a pronounced peak for formation of β phase still exists. These DSC results reveal main effects of processing temperature on precipitation: (1) ECAP processing at RT or 200 °C have litter effect on the precipitation sequence; (2) processing at high temperature accelerate decomposition kinetics of the alloy. Such accelerated behavior has been observed in AA6060 processed by HPT [9].

**Figure 7:**
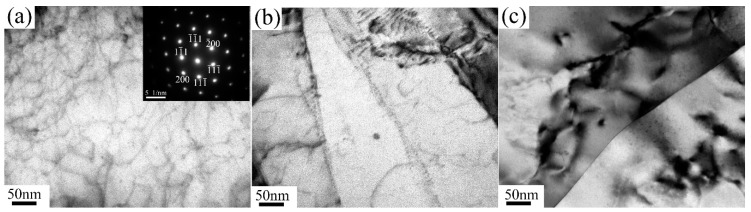
Bright-field images (the zone axis B = [110]) of the alloy processed at RT: (**a**) one pass; (**b**) two passes; (**c**) eight passes.

**Figure 8:**
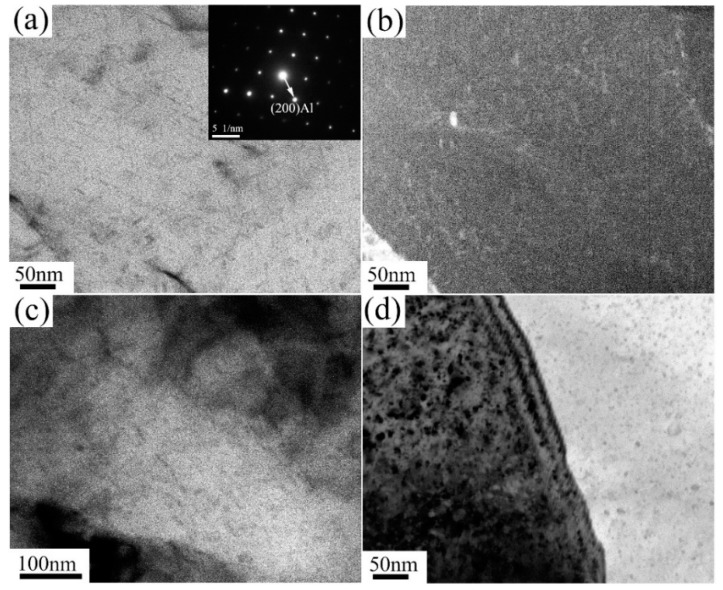
TEM micrographs for the alloy processed at 200 °C: (**a**) bright-field image after one pass (B = [110]) (insert), (**b**) dark-field image after one pass, (**c**) bright-field image after two passes, and (**d**) bright-field image after eight passes.

**Figure 9:**
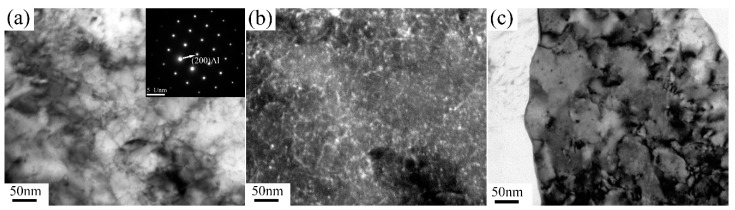
TEM micrographs of the alloy processed on TST condition: (**a**) bright-field image after two passes (B = [110]) (insert); (**b**) dark-field image after two passes; (**c**) bright-field image after eight passes.

Figure 7, Figure 8 and Figure 9 show the evolution of precipitates with pass numbers for 6063 alloy processed at RT, 200 °C and TST conditions. There are limited precipitates in samples processed at RT from one pass to eight passes. Many dots in micrographs could be due to the element enrichment. However, a large density of needle-like and rod-like nanoscaled precipitates with diameter of only several nanometers and extreme length beyond 30 nm appear at 200 °C condition, as shown in Figure 8a. Their typical shape and size are quite consistent with these of needle-like β’’ and rod-like β’ observed in the literature [17]. Thus, it is reasonable to assume that these precipitates are β’’ and β’. After eight passes at 200 °C, a high density of slightly coarse phases ranged from 2 nm to 7 nm (Figure 8d) and some large precipitates with diameter of ~20 nm (as marked in Figure 5f) are visible in the grain interior. These large precipitates can be identified as β’/β [7,27]. Clearly, these β’’ and β’ precipitates at 200 °C condition can be confirmed from DSC plots for the samples processed by one pass and eight passes at 200 °C, which indicate that β’’ and β’ have precipitated from the matrix during processing. In the samples processed in TST condition, as shown in Figure 9, nanosized precipitates are clearly observed in the vicinity of the high density of dislocations. These precipitates exhibit a globular shape with a typical size of only ~2 nm and may be precursors of β-Mg_2_Si due to the fact that their size is smaller than the size of typical β-Mg_2_Si with a few tens of nanometers [7].

### 3.3. Mechanical Properties and Electrical Conductivity of the 6063 Al Alloy After ECAP Processing

**Figure 10:**
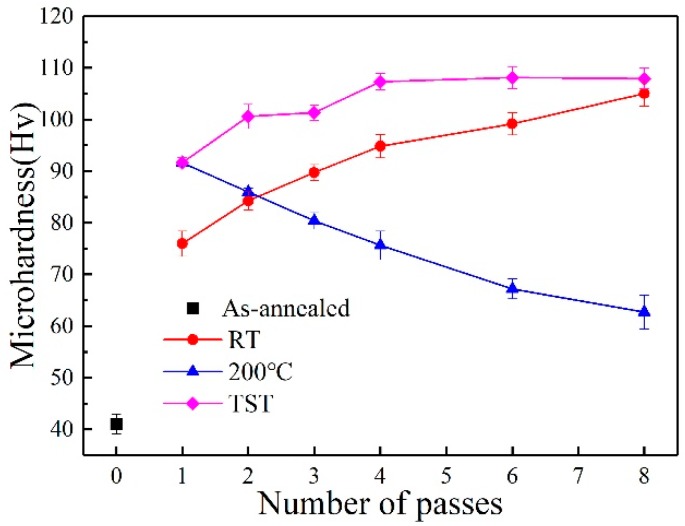
Evolution of microhardness with pass numbers in the alloy processed by ECAP at different conditions.

The Vicker-hardness of 6063 alloy processed on different conditions is plotted as a function with the pass number in Figure 10. Processing at 200 °C condition leads to a decrease in hardness with increasing pass number. By contrast, processing at RT and TST conditions provide the gradual improvement of hardness with increasing pass number. After one pass, the hardness increases with a sharp rise from 41 Hv of the annealed state up to 76 Hv of RT condition and 92 Hv of 200 °C condition. Sample processed by one pass at 200 °C receive the higher value of hardness than that at RT condition (~22% increasement). After two passes, the sample processed at TST condition obtains extremely higher hardness value (~101 Hv) than that on the other two conditions. The hardness value is comparable to that of sample processed by six passes at RT condition (~99 Hv). After eight passes, sample processed at TST condition have the highest hardness of 108 Hv among the three conditions.

**Figure 11:**
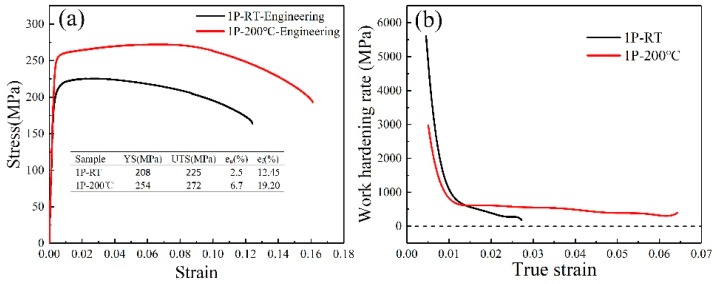
(**a**) Tensile engineering curves of the alloy processed after one pass at RT and 200 °C; (**b**) work-hardening rate as a function of true strain. Yield, ultimate tensile strength, uniform elongation (e_u_) and total elongation (e_f_) are reported in the graph insert.

Figure 11a presents the typical tensile engineering curves of samples processed by only one pass at RT and 200 °C. It can be clearly seen that strength and uniform elongation of sample processed by one pass at 200 °C condition are improved compared with these at RT condition. The improved yield strength and uniform elongation are about 22% and 168%, respectively, as shown in Figure 11a. Figure 11b shows work-hardening rate plotted versus true strain of the samples subjected to one-pass processing at RT and 200 °C. As can been seen from Figure 11b, after sharp decrease stage about 1.5% strain, the value of work-hardening rate of sample processed at 200 °C is higher than that at RT condition. Moreover, there is still a noticeable plateau to higher strain for sample processed at 200 °C, demonstrating that one-pass processing at 200 °C introduces better work-hardening ability than that at RT. This agrees with the higher uniform elongation for 200 °C condition in the engineering stress-strain curves (Figure 11a). It suggests that after severe plastic deformation, the uniform elongation is primally controlled by the work-hardening rate due to low strain-rate sensitivity.

**Figure 12:**
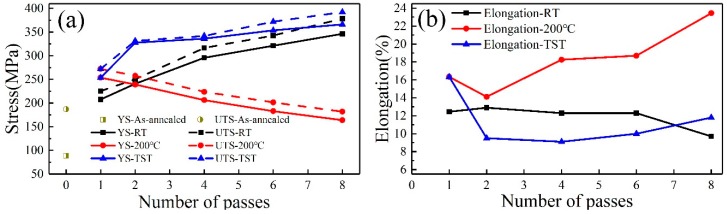
Tensile properties of the alloy processed by ECAP processing on different condition: (**a**) yield and ultimate tensile strength; (**b**) total elongation.

The tensile properties of 6063 alloy subjected to processing at different conditions are shown in Figure 12, where the datum points at zero pass come from tensile testing of the as-annealed 6063 alloy before ECAP processing. As exhibited in Figure 12, both strain and temperature have significant effects on tensile properties. In the three processing conditions, only single pass leads to a dramatic jump of yield strength (from the 88.5 MPa up to the range of 208 MPa to 254 MPa) and then a higher level of deformation induces gradual increase, excepted at 200 °C condition. After two passes, the improvement of yield strength about ~36% is achieved in sample processed in TST condition as compared with that in RT condition. Then, after eight passes, the highest strength (366 MPa) is reached for sample in TST condition, which is ~20 MPa higher than that in RT condition.

**Figure 13:**
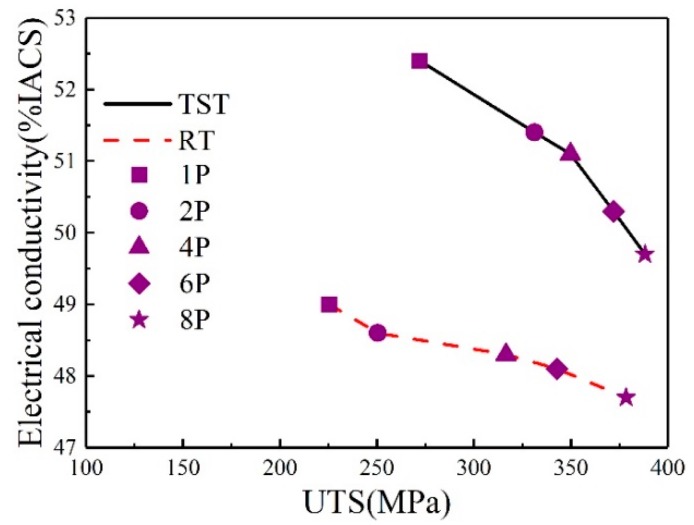
Electrical conductivity of the alloy processed by ECAP processing as a function of ultimate tensile strength.

As shown in Figure 13, the electrical conductivity is also sensitive to both processing temperature and strain. At RT and TST conditions, the electrical conductivity decreases with increasing amount of strain. For the samples in TST condition, electrical conductivity is in the range of 52.4% IACS to 49.7% IACS and the counterpart ultimate tensile stress is from 272 MPa to 392 MPa, respectively. For the samples in RT condition, electrical conductivity is in the range of 49% IACS to 47.7% IACS and the counterpart ultimate tensile stress is from 225 MPa to 378 MPa, respectively. After two passes, processing in TST condition leads to a better combination of strengths and electrical conductivity (~51.4% IACS and 331 MPa) than that in RT condition (~48.6% IACS and 250 MPa).

## 4. Discussion

### 4.1. Influence of ECAP Processing Route on Microstructures

Processing temperature and strain induce by successive pressing have significant effects on the microstructures as observed in the present study. Grain refinement of the sample processed at 200 °C is very different from that at the other two conditions. The sample processed by eight passes at 200 °C exhibits a larger average grain size than that at RT and TST conditions. It is expected that a higher dynamic recovery rate is obtained at higher temperature. In addition, the over-decomposition of the solid–solution for samples processed at 200 °C condition, as confirmed by a coexistence of large density of fine precipitates and lager precipitates in the grain interior, can reduce the solute atoms segregated at grain boundaries, which leads to the grain boundaries being more mobile and thus grain coarsening at 200 °C [9], as shown in Figure 5f.

In comparison with RT condition, pronounced β’’and β’ were observed in the samples processed at 200 °C. In the case of these precipitates at 200 °C condition, the rapid decomposition of solid–solution at elevated processing temperature is likely a dominant factor [6]. Moreover, it should be noted that the fluctuations of size and distribution of precipitates occurred, especially for the sample processed by eight passes at 200 °C. The defects induced by processing play an important role in nucleation [17,28] and growth of precipitates. It has been observed that dislocations can accelerate the formation of precipitates by serving as fast diffusion paths for solute atoms in 6063 alloy during ECAP at RT [29], in 6061 and 6069 alloys during ECAP at 100 °C and 170 °C [30], and in Al 7136 alloy during ECAP at 200 °C [31]. Under TST condition, needle-like β’’ and rod-like β’ for the one-pass sample processed at 200 °C no longer appear in the samples processed by following successive passes at RT. Indeed, the fragmentation of precipitates during ECAP processing and dissolution of partial precipitate fragments by intense shear deformation have been observed in Al-Mg-Si alloys [10]. The globular-like nano-precipitates in TST condition are probably from fragmentation of these β’’ and β’ due to smaller size than that of needle-like β’’ and rod-like β’or from formation of new precursors of β-mg_2_si.

### 4.2. Strengthening 6063 Alloy by One-Pass ECAP Processing

The yield strength of the 6063 alloy processed after one pass could be mainly attributed to four mechanisms: grain boundary strengthening, solid–solution strengthening, dislocation strengthening, and precipitate strengthening. The yield strength can therefore be expressed as following [32]:

(1) $$\sigma_{S}=\sigma_{0}+\Delta \sigma_{GB}+\Delta \sigma_{dis}+\Delta \sigma_{SS}+\Delta \sigma_{P}$$

where $\sigma_{0}$, $\Delta \sigma_{GB}$, $\Delta \sigma_{dis}$, $\Delta \sigma_{SS}$ and $\Delta \sigma_{P}$ are contributions from lattice friction, grain boundaries, dislocations, elements in solid–solution, and precipitates, respectively. When considering pure Al, the friction stress $\sigma_{0}=10\mathrm{MPa}$ [33]. For Al alloys with grain size ranged from micrometer to submicrometer scale, it has been demonstrated that grain boundary strengthening can be expressed by the Hall–Petch relationship [34]. In this case, the influence of grain boundary on the yield strength of samples processed after one pass in both conditions might be estimated using the following equation:

(2) $$\Delta \sigma_{GB}=K_{y}d^{-1/2}$$

where $k_{y}=0.12\mathrm{MPa}/\sqrt{m}$ [35]. For samples processed one-pass ECAP at RT and 200 °C, the contribution of grain boundary to the yield strength is estimated about 16 MPa. As discussed in the literature [7], the upper bound of dislocation density are estimated as 10^14^ m^−2^. The strength increment from dislocation strengthening can be estimated by the flowing relationship:

(3) $$\Delta \sigma_{dis}=aMGb\rho^{1/2}$$

where a is a dimensionless geometrical constant (a=0.3), M is dimensionless mean orientation factor for the fcc polycrystalline matrix (M=3.06), G is shear modulus for fcc Al (G=26.9GPa), b is the magnitude Burgers vector (b=0.29nm). According to Equation (3), the contribution of dislocations to the yield strength $\Delta \sigma_{dis}$ is estimated about 72 MPa. The influence of solid hardening on the strength is given by [36]:

(4) $$\sigma_{i}=k_{i}c_{i}{}^{2/3}$$

where $\sigma_{i}$ is the absolute increase in yield stress from the solute i, $c_{i}$ is the concentration (in weight percent) of the i solute, and $k_{i}$ is the a scaling factor for the i solute. For the Mg and Si atoms, the values of the scaling factor are 29 and 66.3 MPa (wt.%)^−2/3^ [36], respectively. The increase in the strength due to solute hardening can be assumed by additive contributions from Mg and Si [7]. The maximum possible solute hardening due to Mg and Si can be estimated by expecting that all of Mg (0.52 wt.%) and Si (0.49 wt.%) are dissolved in the Al matrix. The maximum contribution of solution hardening to the yield strength is estimated about 60 MPa. Based on the above, the strengthening contribution of the nanosized precipitates can be calculated. For the sample processed by one pass at 200 °C, $\Delta \sigma_{P}=\sigma_{S}-\sigma_{0}-\Delta \sigma_{GB}-\Delta \sigma_{dis}-\Delta \sigma_{SS}$. The calculated value of ~97 MPa is a lower limit, and thus nanosized precipitates strengthening play a dominant role for the increase in strength of the sample processed by one pass at 200 °C.

The work-hardening rates of samples processed by one-pass ECAP in two conditions are shown in Figure 11b. Generally, work-hardening rate is controlled by a dynamic competition process: dislocations generation due to deformation and dislocations annihilation due to dynamic recovery [37]. The dynamic recovery of deformed alloys is controlled by the climb and cross-slip of dislocations. The nano-precipitates can act as barriers to moving dislocations and thus have a significant effect on dislocations accumulation [38]. The observed apparent high work-hardening rate of sample processed by one-pass ECAP at 200 °C should, at least partially, be attributed to dislocations accumulation in the presence of high-density nano-precipitates, which can hinder dynamic recovery effect. This observation is somewhat different with the previously published literature in an Al 6063 alloy subjected to cryrolling at large strain where the solutes in solid–solution exhibited a stronger effect on work hardening of the alloy during cryorolling than presence of precipitates in peak-aged state [39]. A possible reason for this difference is that the size of precipitates from static peak aging is slight larger than these nano-precipitates in sample processed by one pass at 200 °C in the present study. Therefore, the pronounced nano-precipitates may lead to a higher work-hardening rate in comparison with the presence of higher number of solid atoms in processed matrix.

### 4.3. Effect of ECAP Processing Temperature on Properties

The mechanical properties of 6063 alloy under different processing conditions were characterized by hardness (Figure 10) and tensile tests (Figure 12). These results reveal that processing at RT leads to an increase in strength with increasing pass number. In contrast, processing at 200 °C leads to a decrease in strength. The yield strength difference between samples processed by eight passes at RT and 200 °C conditions reaches 182 MPa, which is ~20 MPa higher than that of sample processed by eight passes at 200 °C. It appears to be associated with the grain refinement from 0.6 µm to 0.08 µm and a low level of strengthening contribution from the relatively coarse globular precipitates, similar to the over-aged state. It has been suggested that coarsening effect may weaken the strengthening effect of precipitates for Al6061 alloy processed by ECAP [10] and for the AA 6060 alloy processed by HPT [9]. The sample processed by two passes at TST condition has an average grain size of ~0.39 µm, lager than ~0.23 µm and ~0.11 µm for the samples processed by two and four passes at RT condition, respectively. However, the strength of sample processed by two passes at TST condition is higher than these of samples processed by two and four passes at RT condition with smaller average grain sizes. This cannot be explained by the Hall–Petch relationship. For the sample processed under TST condition, the most significant structure features are nanoscaled precipitates and high density of dislocations. Therefore, in addition to grain refinement, high dislocation density (Figure 5) and nanoscaled precipitates (Figure 9) provide the sample processed in TST condition with dominant strengthening effects. Comparison with RT and 200 °C conditions, high strength obtained by samples in TST condition could be attributed to two main factors: (1) low processing temperature followed by high processing temperature tends to decrease grain sizes; (2) the nano-precipitates can strongly impede dislocations movement, which may lead to a higher strength than that are based on solid–solution hardening. Thus, the samples processed in TST condition can promptly obtain higher strengths than these in the other two conditions. Moreover, a decrease in content of solutes due to formation of nano-precipitates plays a positive role in electronical property.

Each strengthening factor can generate distortion in crystal lattice structure and lead to degradation of electrical conductivity because of electrons scattering from these additional distortions. According to the Mattissen–Flemming rule, the electrical resistance might be affected by vacancies, dislocations, grain boundaries, solute elements dissolved in the matrix and second-phase precipitates. Moreover, electrical conductivity is extremely sensitive to the solution elements in aluminum matrix and second-phase precipitates in similar alloys [19]. It should be noted that several studies [7,19] have shown a normal phenomenon: higher processing temperature can improve the electrical conductivity but it is accompanied by a drop of ultimate tensile strength. However, in the present study, it is noted that a better combination of properties was achieved by sample subjected to processing in TST condition. From results of DSC (Figure 6) and TEM (Figure 7), it can be confirmed that processing in RT condition may lead to a low level of decomposition of solid–solution as compared to that in TST condition. Since solute atoms is more effective in scattering electrons as compared with precipitates [14,40], high-density nano-precipitates in sample processed in TST condition can minimize the negative effect of solute atoms on the electrical conductivity. As a result, samples processed in TST condition have higher electrical conductivity than that of samples processed in RT condition.

## 5. Conclusions

In the present work, the effect of ECAP processing temperature on the microstructures, mechanical and electrical properties were investigated in 6063 aluminum alloy, and the major findings are summarized as follows:

(1) Both grain refinement and precipitation are extremely dependent on ECAP processing temperature and strain. Of the three processing conditions, only processing at 200 °C produced a decrease of strength with increasing pass numbers, probably because of coarsening effects of grain and precipitate.
(2) Formation of ultrafine-grained structure and nano-scale precipitates due to SPD by ECAP processing in TST condition play a key role in an increase in strength of the alloy. In particular, processing in TST condition is characterized by lower amount of strain required for high strength compared to RT condition (two passes in TST condition, comparable to six passes in RT condition).
(3) Electric conductivity of processed alloy is highly sensitive to ECAP processing temperature. It was confirmed that the pronounced precipitates in the alloy processed under TST condition also have a positive effect on electrical conductivity. Finally, this work shows that TST schedule during ECAP processing can be considered to be an effective approach to getting ultrafine grains with nano-scale precipitates and hence optimizing the combination of improved strength and electricity conductivity in heat-treatable Al alloys.
